# Acute pain assessment and management in the prehospital setting, in the Western Cape, South Africa: a knowledge, attitudes and practices survey

**DOI:** 10.1186/s12873-020-00315-0

**Published:** 2020-04-28

**Authors:** Andrit Lourens, Peter Hodkinson, Romy Parker

**Affiliations:** 1 Division of Emergency Medicine, University of Cape Town (UCT), Cape Town, South Africa; 2 Department of Anaesthesia and Perioperative Medicine, University of Cape Town (UCT), Cape Town, South Africa

**Keywords:** Prehospital, Acute pain assessment and management, Analgesia, Knowledge, attitudes and practices

## Abstract

**Background:**

Acute pain is frequently encountered in the prehospital setting, and therefore, a fundamental aspect of quality emergency care. Research has shown a positive association between healthcare providers’ knowledge of, and attitudes towards pain and pain management practices. This study aimed to describe the knowledge, attitudes, and practices of emergency care providers regarding acute pain assessment and management in the prehospital setting, in the Western Cape, South Africa. The specific objectives were to, identify gaps in pain knowledge; assess attitudes regarding pain assessment and management; describe pain assessment and management behaviours and practices; and identify barriers to and enablers of pain care.

**Methods:**

A web-based descriptive cross-sectional survey was conducted among emergency care providers of all qualifications, using a face-validated Knowledge, Attitudes and Practices of Pain survey.

**Results:**

Responses of 100 participants were included in the analysis. The survey response rate could not be calculated. The mean age of respondents was 34.74 (SD 8.13) years and the mean years’ experience 10.02 (SD 6.47). Most respondents were male (69%), employed in the public/government sector (93%) as operational practitioners (85%) with 54% of respondents having attended medical education on pain care in the last 2 years. The mean percentage for knowledge and attitudes regarding pain among emergency care providers was 58.01% (SD 15.66) with gaps identified in various aspects of pain and pain care. Practitioners with higher qualifications, more years’ experience and those who did not attend medical education on pain, achieved higher scores. Alcohol and drug use by patients were the most selected barrier to pain care while the availability of higher qualified practitioners was the most selected enabler. When asked to record pain scores, practitioners were less inclined to assign scores which were self-reported by the patients in the case scenarios. The participant dropout rate was 35%.

**Conclusion:**

Our results suggest that there is suboptimal knowledge and attitudes regarding pain among emergency care providers in the Western Cape, South Africa. Gaps in pain knowledge, attitudes and practices were identified. Some barriers and enablers of pain care in the South African prehospital setting were identified but further research is indicated.

## Background

Acute pain prevalence in the prehospital arena is thought to be high with the assessment and management thereof widely shown to be insufficient at large [1–4]. The South African prehospital setting appears to be no different with two recent studies showing limited evidence of pain assessment, and pain management likely being ineffective [5, 6]. Very little is known about acute pain in the African prehospital setting [7]. In low- and middle-income countries, inadequate pain management is often attributed to a lack of resources and knowledge, poor pain assessment and/or pain being a low priority [8, 9]. Benefits of alleviating acute pain are numerous. Suffering, recovery time, infection risk and the risk for chronic pain are reduced while diagnostic and treatment processes are enabled and patient satisfaction and patient outcomes are improved [10–13]. Evidence also suggests that prehospital analgesia reduces the time to administration and likely increases appropriate subsequent emergency department analgesia [13, 14]. Pain management is a fundamental aspect of quality prehospital care, and despite apparently straightforward approaches, in theory, it is extremely challenging to achieve, even in well-developed systems [15–17].

Various barriers to prehospital pain management like lack of knowledge, pain assessment challenges, language barriers, organisational culture, pain underestimation and practitioners beliefs and attitudes have been highlighted [13, 18–21]. Children are less likely to have pain assessed and managed [22–25] and females regardless of age and pain severity less likely to received opioids [25–28] while patients in severe pain and those spending more time with Emergency Medical Services (EMS) more likely to receive opioids [28, 29]. Some prehospital practitioners express an attitude that pain is not life-threatening, therefore, a minor priority [18, 19]. Male prehospital practitioners express more enduring (stoic) viewpoints regarding the need for analgesia while older practitioners have more negative attitudes about assessing pain medication requirements [30]. Moreover, prehospital providers from various high-income countries (HIC) still report that pain assessment and management during undergraduate studies receive limited focus [18, 20, 21] and that continuous pain education is lacking [22].

Knowledge, attitudes, and practices (KAP) surveys can be conducted to measure what is known about a health problem, develop a baseline understanding of beliefs and behaviours, and even to quantify change after health interventions [31, 32]. This study aimed to describe the KAP of emergency care providers regarding acute pain assessment and management in the prehospital setting, in the Western Cape (WC), South Africa (SA). The specific objectives were to, identify gaps in pain knowledge; assess attitudes regarding pain assessment and management; describe pain assessment and management behaviours and practices; and identify barriers to and enablers of pain care.

## Methods

### Study design

A web-based [33] descriptive cross-sectional KAP in Pain survey was conducted among prehospital emergency care providers of all qualifications, registered with the Health Professionals Council of South Africa (HPCSA) and currently practising in the WC, SA.

### Study setting

Respondents to this study were emergency care providers from the WC province, one of nine provinces in SA with a population of more than 6.3 million people, which accounts for 11.3% of the SA population. The WC is sub-divided into six districts, one large metropolitan area with a well-developed healthcare network including several tertiary and many district-sized hospitals (the City of Cape Town), and five (rural or peri-urban areas) districts (Cape Winelands; Overberg; West Coast; Eden (or Garden Route) and Central Karoo districts) characterised by largely small district or regional hospitals separated by long distances [34]. Most of the communities in the WC are served by the public (government-operated) EMS system while various private ambulance services deliver a service to the minority of the population who can afford medical insurance.

Emergency care education in SA is broadly categorised into basic (BLS), intermediate (ILS) or advanced life support (ALS) level qualifications which evolved from a three-tiered short course framework to more professional tertiary (undergraduate) level qualifications in recent years [35, 36]. At the time of the study, non-ALS practitioners’ scope of practice limited their analgesic options to inhaled nitrous oxide (Entonox®), which is regularly not available on most ambulances in the WC. For these practitioners (the majority of the workforce [35]), to deliver pain relief or to provide stronger analgesia, a request for assistance from a higher (ALS) qualified practitioner, who is able to administer intravenous analgesia (morphine or ketamine), needs to be made and the availability of these practitioners is often limited.

### Sampling and sample size

A non-probability, convenience sampling strategy was utilised, with the aim to obtain a representative sample of each level of qualification within the target population. Based on the number of emergency care providers (9091) registered under the different HPCSA (iRegister) [37] emergency care registers in the WC, a sample size of 192 was calculated using an online sample size calculator [38] with a 7% margin of error in survey responses, a 95% confidence interval (CI) and a 50% response distribution. The actual sample size obtained was 100 respondents. With this sample, the margin of error in survey responses was 9.75% with a 95% CI and a 50% response distribution.

### Data collection

The development of the questionnaire was based primarily on two existing surveys - the Knowledge and Attitudes Survey Regarding Pain (KASRP) used to assess nurses and other healthcare providers (HCPs) (revised 2014) [39] and the Pediatric Nurses’ Knowledge and Attitudes Survey Regarding Pain [40] as well as including questions adapted from the article by Pocock [41] and questions specific to the SA prehospital setting. Dependent on the level of qualification, emergency care providers practice within a set scope with certain medication limitations, therefore, questions related to pharmacological pain management were restricted. Three experts (including an expert in pain management) made comments and suggestions on the structure and length of the questionnaire, appropriateness of the questions, accuracy of answers and response options after which the survey was piloted among emergency care providers. Remarks received during the pilot study were mostly related to the length of the questionnaire. The questionnaire was, therefore, refined to include the questions/statements most appropriate and relevant to the setting. The questionnaire consisted predominantly of closed-ended questions with limited open-ended questions in six sections including demographic questions; “true/false/don’t know” statements (18); likert scale statements (8); multiple-choice questions (MCQs) (5); barriers and enablers (selection from the list provided); and two case studies (measuring pain assessment and management practices (free text questions)) (Additional file 1).

A recruitment flyer containing an embedded link and quick response (QR) code to the online survey was sent to senior management of the different EMS systems for distribution to staff members. Data collection started on the 11th of October 2018 and was extended due to poor participation until the 31st of March 2019. The management structure of the services involved was requested to remind staff of the survey in December 2018 and January 2019. All completed questionnaires were anonymised by the web-based survey service [33].

### Data analysis

Data were analysed using SPSS Statistics, Version 25 [42]. The primary outcome of the study was knowledge and attitudes regarding pain scores and percentages with secondary outcomes being factors influencing scores, gaps in pain knowledge, attitudes and practices, the proportion of selected barriers and enablers of pain assessment and management in the prehospital setting and the description of pain management practices. The overall score was calculated by adding the scores obtained for the true/false/don’t know statements, likert scale statements and MCQs. For the true/false/don’t know statements, 1 score was assigned for a correct response and 0 for incorrect or don’t know responses. The three-point Likert scales were collapsed into dichotomous variables (correct and incorrect). A correct response to a statement was assigned a score of 1 while 0 was assigned to an incorrect or neutral response. Descriptive statistics were used to express the results and tables used to present demographic information (frequencies, percentages, means, standard deviation and ranges), survey responses (frequencies and percentages), overall scores (means, standard deviation, ranges and 95% CI) and selected barriers and enablers of pain assessment and management (frequencies and percentages). Shapiro-Wilk tests were conducted to assess normality in the data. To determine whether scores correlated with demographic information Spearman’s correlation coefficient were conducted. To identify whether demographic information may influence overall scores, the non-parametric tests, Mann-Whitney U test and Kruskal-Wallis H test were conducted. For the case scenarios, self-reported pain scores for each case were reported through descriptive statistics (frequencies, percentages and medians) while free-text responses to the open-ended question related to the management of the two cases were summarised in a table. The developers of the KASRP survey [39] recommended that distinguishing between knowledge and attitude items during data analysis be avoided.

## Results

### Participation

**Fig. 1:**
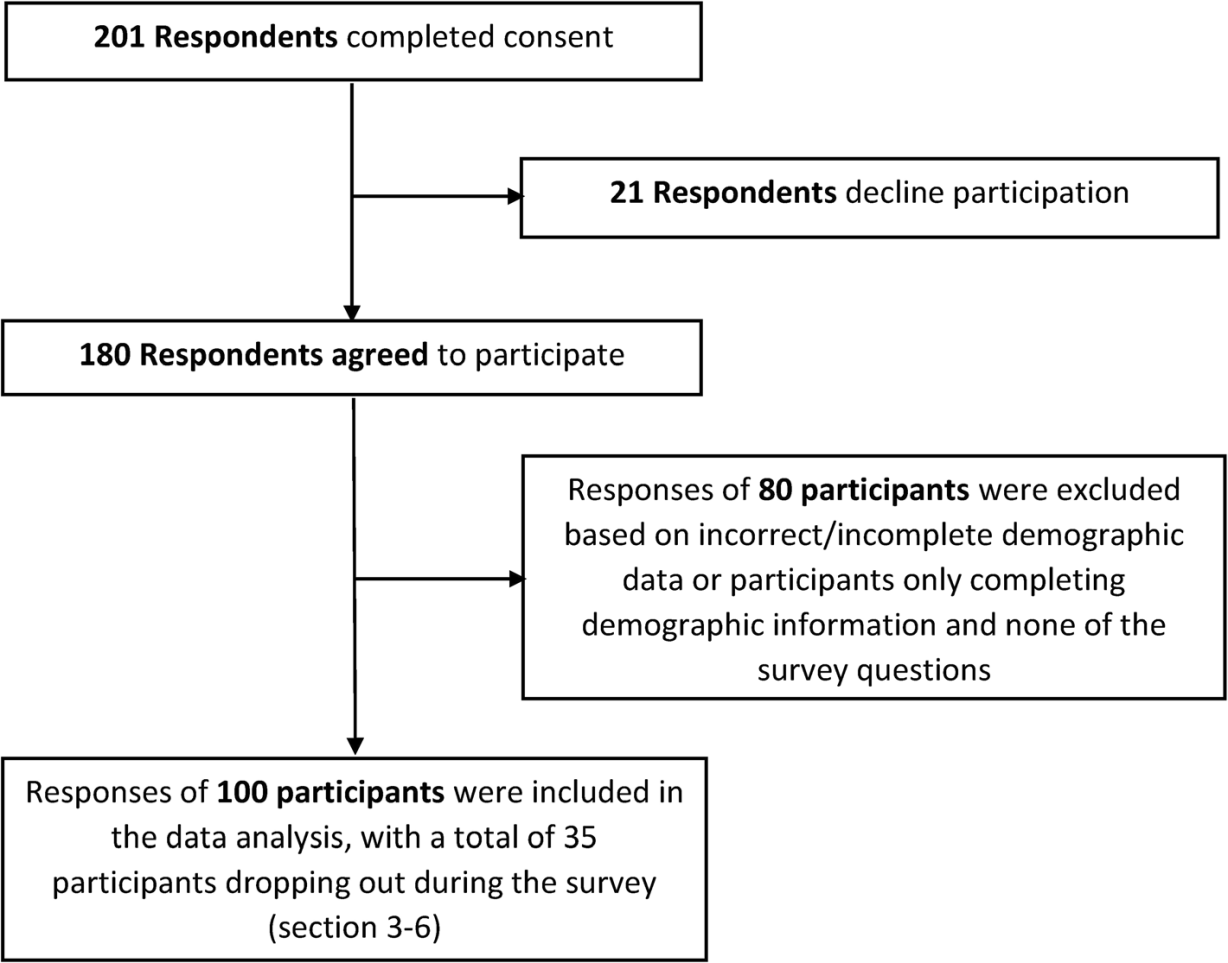
Flow diagram of survey participation

Figure 1 presents a flow diagram of survey participation and the number of responses included in the data analysis. A relatively new South African law, the Protection of Personal Information (POPI) Act 4 of 2013 [43], protects South Africans’ right to privacy and restricts access to personal information. Consequently, the organisations which approved the research distributed the questionnaire internally. The number of individuals to which the questionnaire was disseminated was unknown, making accurately calculating the survey response rate unanticipatedly difficult.

### Demographic information (section 1)

The mean age of respondents was 34.74 (SD 8.13) years and ranged between 21 and 57 years, while years of experience ranged between 1 and 29 with a mean of 10.02 (SD 6.47) years (see Table 1).

**Table 1:** Demographic characteristics of respondents (*n* = 100)

Gender:	n (%)
Male	69 (69%)
Female	31 (31%)
Level of qualification:	n (%)
Basic Life Support (BLS)^a^	20 (20%)
Intermediate Life Support (ILS)^b^	48 (48%)
Advanced Life Support (ALS)^c^	32 (32%)
Region of employment:	n (%)
Cape Town Metropolitan	29 (29%)
Cape Winelands District	8 (8%)
Central Karoo District	8 (8%)
Eden District	41 (41%)
Overberg District	8 (8%)
West Coast District	6 (6%)
Years’ experience (range):	n (%)
0–10 Years	60 (60%)
11–20 Years	32 (32%)
21–30 Years	8 (8%)
Current role within EMS	n (%)
Operational Emergency Care Provider	85 (85%)
Other^d^	15 (15%)
Continuing medical education on acute pain assessment and management received in the last 2 years	n (%)
Yes	54 (54%)
No	46 (46%)
Sector of employment:	n (%)
Public/Government Sector	93 (93%)
Private Sector	7 (7%)
Age groups:	n (%)
21–30 Years	38 (38%)
31–40 Years	40 (40%)
41–50 Years	19 (19%)
51–60 Years	3 (3%)

Footnote: ^a^ Include the Basic Ambulance Assistant (BAA) qualification, ^b^ Include the Ambulance Emergency Assistant (AEA) qualification, ^c^ Include the following qualifications: Emergency Care Technician (ECT), Critical Care Assistant (CCA) paramedic, National Diploma in Emergency Medical Care (NDEMC) paramedic, Emergency Care Practitioner (ECP), ^d^ Include the following roles: Supervisor/Manager, Higher education, Rescue, CQI/Patient safety, Emergency Medical Care Student and Emergency Medical Services Volunteer

### Knowledge and attitudes regarding pain management in the SA prehospital setting (sections 2, 3 and 4)

For the “true/false/don’t know” section (2) of the questionnaire, scores (*n* = 100) ranged between 3 (17%) and 18 (100%) with a mean score of 10.14 out of 18 or 56.38% (SD 17.02, 95%CI 53.00–59.76). Frequencies and percentages of correct responses for the true/false/don’t know statements are reported in Table 2. Eighty-three percent of respondents correctly indicated that self-reported pain using the numeric rating scale is the quickest way to assess pain, while 41% wrongly believed that giving patients sterile water by injection (placebo) is a useful test to determine if the patient’s pain is real. Only 25% of respondents were aware that the patient’s culture and/or spiritual beliefs influenced the experience and expression of pain while only 31 and 29% were respectively aware that vital signs and patient behaviour are poor/unreliable indicators of pain severity.

**Table 2:** Frequencies and percentages of correct responses for “true/false/don’t know” section (*n* = 100)

True/false/don’t know statements	n (%)
Pain can be defined as “an unpleasant sensory and emotional experience associated with actual or potential tissue damage or described in terms of such damage” (True)*.	90 (90%)
Non-pharmacological methods, such as splinting, are effective methods to assist pain relief **(True).**	86 (86%)
In the event that a patient’s pain is not managed, their overall clinical condition may deteriorate (progressively worse) **(True)**.	84 (84%)
Self-reports of pain according to the numeric rating scale (pain assessment tool) are the quickest way to assess pain **(True).**	83 (83%)
Entonox® (Nitrous Oxide) is a potent analgesic with a very rapid onset of action and is quickly eliminated from the body **(True).**	82 (82%)
Children younger than 11 years cannot reliably report pain, therefore, clinicians should rely solely on the parent’s assessment of the child’s pain intensity **(False).**	75 (75%)
Similar or comparable stimuli, in different people, will produce the same intensity or severity of pain **(False).**	65 (65%)
If you do not consider the condition to be painful the patient should not receive analgesia (pain relief) **(False).**	61 (61%)
In the pre-hospital environment, patients should not receive analgesia for chronic medical conditions **(False).**	61 (61%)
Giving patients’ sterile water by injection (placebo) is a useful test to determine if their pain is real **(False).**	59 (59%)
Unconscious patients do not experience pain **(False).**^a^	53 (53%)
Due to an underdeveloped nervous system, children younger than 2 years, have decreased sensitivity to pain and limited memory of painful experiences **(False).**	39 (39%)
Adult and paediatric patients who can be distracted from their pain are usually not experiencing severe pain **(False).**	39 (39%)
Vital signs are always reliable (good) indicators of the intensity or severity of a patient’s pain **(False).**	31 (31%)
Young infants, less than 6 months of age, cannot tolerate opioids/narcotics (like morphine) for pain relief **(False).**	30 (30%)
Patient behaviour is a more reliable (good) indicator of pain than a patient’s self-report **(False).**	29 (29%)
The experience and expression of pain are influenced by a patient’s culture and/or spiritual beliefs **(True).**	25 (25%)
If the source of a patient’s pain is unknown, opioids/narcotics (like morphine) should not be used during the pain evaluation period, as this could mask the ability to correctly diagnose the cause of pain **(False).**	23 (23%)

*Correct responses for each statement indicated in **bold**

^a^ There is a debate in the literature that pain is a construct of the conscious brain and all other processes contributing to pain should be referred to as nociception. Based on such an understanding, pain cannot be felt by an unconscious person. However, the curricula of EM practitioners in South Africa refer to pain pathways and pain processes at both the unconscious and conscious levels of the nervous system without discriminating between pain and nociception. Hence, in this context, this statement is regarded as false

Ninety-one (91%) of the 100 respondents completed the 3-point Likert-scale section (3). Correct responses ranged between 0 (0%) and 7 (100%) out of 7 with an average percentage of 64.68% (SD 22.87, 95%CI 59.92–69.44). The correct responses for the Likert statements are depicted and ranked in Table 3. Only 33% of respondents disagreed that their experience dealing with patients in pain allows them to score patients’ pain more accurately than the patient themselves and 62.6% disagreed that parents or guardians of children should not be present during painful procedures.

**Table 3:** Frequencies and percentages of correct responses for Likert-scale section (*n* = 91)

Likert-scale statements	n (%)
Using a pain assessment tool is a necessary instrument in pain assessment and pain management decision making (Agree)*.	76 (83.5%)
Patients should not be included in the pain management decision-making process **(Disagree).**	75 (82.4%)
The main reason for administering analgesia (pain relief) is to enable the patient to get to the ambulance **(Disagree).**	73 (80.2%)
It is better to be stoic (endure pain or hardship without showing their feelings or complaining) about pain than totally open about it **(Disagree).**	60 (65.9%)
Parents or guardians of children should not be present during painful procedures **(Disagree).**	57 (62.6%)
Expectations of my peers or the company/EMS service I work for, strongly influence my pain management practice **(Disagree).**	41 (45.1%)
I believe that my prior experience dealing with patients in pain allows me to score patients’ pain more accurately than the patient themselves **(Disagree).**	30 (33.0%)

*Correct responses for each statement indicated in **bold**

Statement 35 of section 3 required respondents to share their own opinion on whether they believe the current HPCSA protocols provide sufficient and appropriate pain management options for the SA prehospital setting. Of the 91 respondents, 46.2% (*n* = 42) disagreed while 14.3% (*n* = 13) neither agreed nor disagreed and 39.6% (*n* = 36) agreed with the statement.

The mean score for the MCQs (see Table 4) section (4) was 2.59 out of 5 or 51.72% (SD 21.03, 95%CI 47.24–56.21) and ranged between 0 (0%) and 5 (100%). For 79.3% of respondents, the patient was the most accurate judge of pain intensity while 65.5% of respondents selected the correct wording of the numeric rating scale. For the 87 (87%) respondents who completed all three sections (2, 3, 4), the mean score was 17.40 out of 30 or 58.01% (SD 15.66, 95%CI 54.67–61.35) with scores ranging between 6 (20.0%) and 29 (96.67%).

**Table 4:** Frequencies and percentages of correct responses for multiple-choice questions (MCQs) section (*n* = 87)

Multiple-choice questions	n (%)
The most accurate judge of the intensity of the patient’s pain is: The patient*.	69 (79.3%)
The correct wording when using the Numeric Rating Scale is: **Can you give your pain a score between 0 & 10 with 0 being no pain and 10 the worst imaginable pain.**	57 (65.5%)
Effective management of acute pain is a fundamental component of: **Quality patient care.**	55 (63.2%)
Pain is believed to play a major part in the activation of the ‘stress’ response to injury, leading to all the below, EXCEPT: **Decreased coagulability.**	24 (27.6%)
With regards to pain, all the following descriptors are applicable EXCEPT: **Always associated with actual tissue damage.**	20 (23.0%)

*Correct responses for each statement indicated in **bold**

### Factors influencing knowledge and attitudes regarding pain in the SA prehospital setting

A significant difference was found in the scores obtained by respondents with different levels of qualification (*H* = 30.79, *p* < 0.001) as well as in the scores of respondents with different number of years of experience (*H* = 9.051, *p* = 0.011) (Additional file 2: Table S1). ALS qualified practitioners obtained higher scores compared to both BLS and ILS qualified practitioners**,** and ILS practitioners obtained higher scores compared to BLS practitioners. The median percentage for ALS practitioners were 76.67% (IQR = 56.67–80.00), 56.67% (IQR = 47.50–66.67) for ILS practitioners and 46.67% (IQR = 40.00–50.00) for BLS practitioners. Respondents with 0–10 years’ experience obtained lower scores compared to respondents with 11–20 years’ experience. The median percentage for respondents with 0–10 years’ experience was 51.67% (IQR = 43.33–64.17) and 60.00% (IQR = 53.33–73.33) for those with 11–20 years’ experience. A weak (0.10–0.39) positive relationship (r_s_ = 0.323, *p* = 0.002, two-tailed) was found between overall scores and years’ experience and a moderate (0.40–0.69) positive relationship (r_s_ = 0.597, *p* < 0.001, two-tailed) between overall scores and level of qualification [44].

Respondents who had not attended any specific training on pain management in the preceding 2 years obtained a statistically significant (*U* = 664.0, *p* = 0.017) higher score compared to those who did. The median percentage for respondents who had not attended any specific training on pain management was 60.00% (IQR = 50.00–75.00) and 53.33% (IQR = 45.83–63.33) for those who did. There was no difference in scores analysed by gender (*U* = 718.5, *p* = 0.327) and age group (*H* = 2.800, *p* = 0.424).

### Barriers to and enablers of pain assessment and management (section 5) (*n* = 73)

The three most selected (from list provided) barriers to pain assessment and management were: alcohol and drug use by patients (*n* = 49, 67.1%); language (*n* = 45, 61.6%); and workload or lack of time (*n* = 44, 58.9%). The three most selected enablers were: the availability of higher qualified emergency care providers (*n* = 54, 74%); the understanding that pain management is important (*n* = 43, 58.9%); and the availability of resources such as medication, disposables, and monitoring equipment and a cooperative patient with 52.1% (*n* = 38), each. The complete list of barriers and enablers, as well as the additional barriers and enablers cited by respondents, are available in Additional file 2, Table S2.

### Case studies (section 6) (*n* = 65)

Two case scenarios (see Table 5) were used to determine pain assessment (pain scale 0–10) and management practices. Of the 65 respondents who completed this section, only 35.4% (*n* = 23) assigned a pain score of 8 as self-reported by the patient (patient 1) presenting with no behavioural indicators of severe pain whereas, for the patient (patient 2) with behavioural indicators of severe pain, 64.6% (*n* = 42) of respondents assigned a pain score of 8 as self-reported (see Additional file 2: Fig. S1 and S2). The median pain score for patient 1 was 5 (IQR 3–8) and 8 (IQR 6–8) for patient 2.

**Table 5:** Case scenarios (*n* = 65)

Patient 1: Andrew Andrew is 25 years old and this is his first day following abdominal surgery. As you enter his room, he smiles and continues talking and joking with his visitor. You are required to transport him to a hospital closer to home. Your assessment reveals the following information: BP = 120/80 mmHg; Heart Rate = 80 bpm; Respiratory Rate = 18 bpm. When questioned about his pain, on a scale of 0 to 10 (0 = no pain/discomfort, 10 = worst pain/discomfort) he rates his pain as 8. Questions: - On the patient care report form, you are required to indicate his pain score. Select the number on the below scale (0–10) that represents your assessment of Andrew’s pain. - Indicate how you will manage Andrew’s pain.	
Patient 2: Robert Robert is 25 years old and this is his first day following abdominal surgery. As you enter his room, he is lying quietly in bed and grimaces as he turns in bed. You are required to transport him to a hospital closer to home. Your assessment reveals the following information: BP = 120/80 mmHg; Heart Rate = 80 bpm; Respiratory rate = 18 bpm. When questioned about his pain, on a scale of 0 to 10 (0 = no pain/discomfort, 10 = worst pain/discomfort) he rates his pain as 8. Questions: - On the patient care report form, you are required to indicate his pain score. Select the number on the below scale (0–10) that represents your assessment of Robert’s pain. - Indicate how you will manage Robert’s pain.	

The pain management indicated by respondents for both patients is summarised per level of qualification in Additional file 2: Table S3. Although both patients self-reported a pain score of 8/10 (severe pain), the pain management strategies provided suggest that respondents will manage a patient with behavioural indicators of severe pain more aggressively with pharmacological agents than a patient without behavioural signs of severe pain. Positive points to highlight were the consideration of requesting pain medication from the referring facility (BLS & ILS) before transportation, providing pain relief before moving the patient and the consideration given to non-pharmacological pain management (make patient comfortable, reposition and continuous reassessment). Points of concern were the administration of placebo to test whether the patient is reporting pain honestly and the fact that overall, the descriptions provided suggested that the patients (specifically patient 1), would have been transported with little to no pain relief.

## Discussion

To our knowledge, this is the first study investigating prehospital acute pain knowledge, attitudes and practices in an African prehospital setting, therefore, the findings will be valuable in terms of making recommendations for pain education and further research.

### Knowledge and attitudes regarding pain

Our findings show that there are significant gaps in knowledge and attitudes regarding pain in this cohort of prehospital providers. Research investigating acute pain KAP in Africa and around the world are more commonly conducted in hospitals among nurses and other HCPs. Given the vast differences between nursing curricula and that of prehospital practitioners in South Africa, variances between the in-hospital and out-of-hospital setting and the fact that the questionnaire used was only face validated, makes direct comparison difficult and limited.

The low scores obtained by the respondents in the present study are similar to those reported in studies conducted among nurses and other HCPs from various countries including the African region [45–57]. Studies from North America [58–60], Norway [61] and Australia [62] found substantially higher (72 to 79%) knowledge and attitudes scores among nurses. Still, these studies recommend targeted pain education to overcome specific areas of knowledge and attitudes deficits along with regular in-service pain education [58, 60, 62]. Research among nurses has shown that knowledge and attitudes regarding pain predict pain management practices, with attitudes contributing more to variances in pain management practices than knowledge [63]. Additionally, adequate pain knowledge and favourable attitudes among nurses also correlate positively with patient satisfaction [58]. Although pain education is paramount to altering attitudes and improving pain knowledge, the opinion of some is that education alone may not suffice [59]. In addition to pain education, organisational culture must promote effective pain management practices, provide leadership and support, encourage a culture of continuous learning and promote interdisciplinary teamwork [59]. Further, the implementation of a continuous quality improvement programme [16, 64] and pain protocols or guidelines as well as removing the need to obtain medical control authorisation [13] have likewise improved the provision of prehospital analgesia.

### Factors influencing knowledge and attitudes regarding pain

Our findings show that the level of qualification is a key factor influencing provider knowledge and attitudes regarding pain. This relationship has been confirmed by many international studies [47, 48, 54, 57, 59, 61, 65, 66]. However, the effect of years of experience on scores is uncertain with many differing findings across studies [53, 54, 58, 59, 61, 65, 66]. As would be thought, prior pain education usually results in higher knowledge and attitudes regarding pain scores [48, 58] yet our findings echoed that of an Ethiopian study by Germossa et al. [46] which showed higher scores amongst those not having attended further pain education.

### Gaps in pain knowledge, attitudes and pain management practices

After contrasting participant responses, gaps in knowledge and attitudes regarding pain were identified. Comprehension of the rudimentary principles of pain, pain physiology, pain assessment, indicators of pain severity and pain management was questionable.

Some respondents believed it to be appropriate to administer sterile water to test whether the pain is real, while some believed that pain relief should not be provided if (in their opinion) the condition is not painful. Mistakenly, vital signs were perceived to be a reliable indicator of pain severity [67] while some respondents believed that their prior experience dealing with patients in pain, allows them to score pain more accurately than patients themselves.

Although most respondents indicated that non-pharmacological approaches to pain management assist pain relief, answers to other statements related to non-pharmacological approaches like distraction and emotional support from parents were less positive. Most were correct with regards to pharmacological pain management, however, more than 70% held the belief that infants aged less than 6 months cannot tolerate opioids (poor performance on this item must be considered in terms of the scope of many practitioners limiting their familiarity with infants and opioids).

Despite strong evidence that culture, ethnicity and spirituality plays a significant role in both pain expression and pain behaviour, making behaviour a poor indicator of pain severity [68], comprehension on the part of survey respondents were poor. These misconceptions were further evident in the case scenarios. Respondents considered behavioural indicators of pain more important than self-reported pain. All of which suggests a lack of trust in patients to accurately self-report pain. Further, pain management practices described by respondents for the case studies suggest that the patients will not receive ideal pain relief during the prehospital phase. The practice of administrating sterile water (placebo) to test whether the pain is real, is questionable and likely a violation of the ethical principles [69].

As mentioned, knowledge deficit and practitioners’ perceptions, beliefs and attitudes are barriers to pain assessment and management frequently highlighted in the literature [13, 18, 21, 22, 70]. The inadequacies of pain knowledge in emergency care providers have been attributed to limited focus during initial training, as well as the lack of continuous pain education [13, 18, 21, 22, 70]. The extent of pain education during the initial training of emergency care providers in SA is hard to gauge and varies between training institutions and level of qualification. Nevertheless, all levels of emergency care providers are qualified to provide analgesia in some form. It is imperative that initial emergency care education in South Africa incorporates the topic of pain with pain capabilities specified to include competency in pain assessment, non-pharmacological and scope-specific pharmacological pain management.

### Educational interventions

The study by Germossa et al. [46] additionally showed a significant increase in the mean percentage (41.4 to 63%) for the KASRP scores obtained by nurses after an educational intervention, suggesting that educational initiatives are effective in improving knowledge and attitudes regarding pain. Surprisingly, similar to our findings, the authors reported that in both the pre- and post-intervention testing, nurses with no previous in-service training in pain obtained significantly higher KASRP scores compared to those who received prior pain education [46]**.** This finding could not be explained due to a lack of further information about the in-service training; however, the authors suggested that nurses can change prior knowledge and attitudes regarding pain by attending pain educational programmes and that further tailored continuous education is needed. The positive effects of educational initiatives on pain care were also reported in the prehospital research by French et al. [71] in 2006. The authors found that although paramedics attended an average of 2.2 h of pain education prior to the educational intervention on prehospital pain care, a significant improvement was found in all features of pain assessment and management after the educational intervention [71].

Respondents in this study who reported receiving training on pain assessment and management as part of continuing medical education also performed more poorly than others. Like, Germossa et al. [46] reported, this finding could not be explained due to a lack of further information. Continuing medical education may occur in an array of formal and informal formats. Various factors could have affected the acquisition and retention of the knowledge respondents received during educational initiatives, such as the extent, content, depth and form of education which were not the focus of the current study. Literature also suggests that knowledge gained from pain education will likely decline over time [72].

The current findings suggest that pain education should focus on all aspects relating to pain in order to improve knowledge and attitudes among emergency care providers in SA and that pain education must be continuous. Further research investigating instructional methodologies and strategies to improve pain knowledge acquisition, reinforcement and retention may be beneficial.

### Barriers and enabler

As elsewhere in the world, language barriers, and alcohol/drug use were identified as key barriers to prehospital pain management [73, 74]. Workload and lack of time with patients appear to be barriers specific to the South African prehospital setting. Public EMS, in particular, have a significant workload burden [75], frequently dealing with more than one patient at a time which may influence the delivery of pain care. Availability of higher qualified emergency care practitioners as the foremost enabler of pain management is also likely specific to the SA prehospital setting and due to the structure of the EMS workforce in SA, pain management limitations in the scopes of practice of different levels of qualifications and resource (medication) limitations. The unavailability of the inhaled analgesic medication, Entonox®, in the SA prehospital setting significantly limits the provision of pain management. It is essential that prehospital providers have access to the resources required to facilitate pain management. Although more than half of the respondents identified that pain management is important, the influence of EMS and emergency department culture and leadership support on pain prioritisation and the provision of pain care in the prehospital setting must not be underestimated or overlooked [19, 20]. Studies investigating barriers and enablers of prehospital pain assessment and management have all occurred in HIC [18–21, 70]. The South African prehospital setting is unique in terms of the various levels of qualification and coinciding limitations in scopes of practice, skillset and experience of ALS practitioners, organisational culture, the threat of violence against EMS staff, workload outweighing resource (ambulance) availability, resource limitations, vast distances to health care in rural areas, lack of universal health coverage and disparities in health care, high trauma burden etc. all which may influence prehospital care. Consequently, research to further investigate and describe the barriers to, and enablers of, pain assessment and management in this environment are essential [76].

### Study limitations

Being the first survey of its kind in the African prehospital setting, this study is an important point of departure for acute pain research. Observational studies have limitations, and in this study, participation was poor despite additional recruitment and extended data collection, which may have left the study underpowered to determine significant relationships between demographic groups. Tracking questionnaire distribution and calculating a response rate was unanticipatedly problematic. In the future, survey response rates will have to be carefully assessed, in light of the POPI act and may also be mitigated through technology assisting better tracking of the number of surveys disseminated by third parties, in an anonymous way.

Non-response bias may have been introduced if the respondents that declined to participate were systematically different from those that agreed or if some eligible participants were not reached [77]**.** The survey suffered a 35% dropout rate by the end which may have been secondary to the length of the survey, technical difficulties, work requirements or a lack of interest. The high dropout rate may have introduced further bias in the results due to the under-representation of certain categories of respondents. Respondents who failed to complete the survey were predominantly male (77.1%), had ≤10 years’ experience (68.6%) and were ILS (45.7%) qualified. The generalisability of these findings is not clear, but we believe that despite the small number of respondents, and limited diversity of respondents in terms of the level of qualification, the role within EMS and the region of origin within the province (which may weigh rural practitioners disproportionately), the findings nevertheless create a foundation towards the understanding of the assessment and management of acute pain in the prehospital setting in SA.

Reporting bias may have originated from participants responding in what they perceive to be a professionally desirable manner, instead of exclusively based on personal beliefs, but we believe this bias was reduced by anonymity of the survey, the wide range of questions in different formats and the case study scenarios. The study findings are further limited by the lack of a validated prehospital knowledge and attitudes survey regarding pain. However, to maximise validity the questionnaire was based on existing validated questionnaires, received expert input and was piloted. Finally, although emergency care providers are required to be fluent in English, it may not be the home language [78] of a significant proportion of respondents leading to the possible misinterpretation of statements or questions answered in the survey.

## Conclusion

Our results suggest suboptimal knowledge and attitudes regarding pain among most emergency care providers in the WC, SA. Further, we identified gaps in pain knowledge, attitudes and practices which can be addressed through sufficient attention during undergraduate education as well as tailored, evidence-based pain educational initiatives and ongoing pain education for qualified practitioners. Future work should focus on describing the impact of educational initiatives on pain care as well as exploring the decline in pain knowledge and attitudes over time and what aspects may influence this decline. Although practitioners indicated some issues which they perceive to be barriers and enablers of prehospital pain assessment and management, additional research is indicated to develop a deeper understanding. EMS systems must promote quality pain care and monitor the effectiveness and efficiency of the pain management practice in the prehospital setting, ensuring feedback to operational staff.

## Supplementary information

**Additional file 1.** Informed consent and Knowledge, Attitudes and Practices of Pain Survey

**Additional file 2: Table S1**: Comparing overall score between demographic groups, **Table S2**: Barriers to and enablers of pain assessment and management, **Fig. S1 and S2**: Pain scores for scenario - patient 1 (Andrew) and 2 (Robert), **Table S3:** Pain management for case scenarios.

## Data Availability

The datasets used and/or analysed during the study are not publicly available but are available from the corresponding author on reasonable request.

## Abbreviations

AEA: Ambulance Emergency Assistant
BAA: Basic Ambulance Assistant
CCA: Critical Care Assistant
CI: Confidence Interval
ECP: Emergency Care Practitioner
ECT: Emergency Care Technician
EMS: Emergency Medical Services
HCP: Healthcare Provider
HPCSA: Health Professionals Council of South Africa
KAP: Knowledge, Attitudes and Practices
KASRP: Knowledge and Attitudes Survey Regarding Pain
MCQs: Multiple Choice Questions
SA: South Africa
WC: Western Cape
