# Supplementary material for: Acute pain assessment and management in the prehospital setting, in the Western Cape, South Africa: a knowledge, attitudes and practices survey
Source: BMC Emerg Med. 2020 Apr 28;20:31. doi: 10.1186/s12873-020-00315-0 (PMC7187518; doi:10.1186/s12873-020-00315-0)
Supplement: Supplementary file 1 — Additional file 1. Informed consent and Knowledge, Attitudes and Practices of Pain Survey [file 12873_2020_315_MOESM1_ESM.pdf]

Additional File 1:

Welcome to the KAP Survey

Dear Emergency Care Provider,

You are invited to part take in a Knowledge, Attitude and Practice (KAP) survey on acute pain assessment and management in the South African pre-hospital setting. This KAP survey forms part of a larger study in fulfilment of a Doctoral of Philosophy (PhD) in Emergency Medicine at the University of Cape Town. The main aim of the overall research project is to develop an in-depth understanding of current acute pain assessment and management practices by Emergency Care Providers in the pre-hospital setting, in the Western Cape, South Africa and make recommendations for improvement initiatives.

The project specifically focuses on acute pain assessment and management in the pre-hospital setting which is an area in dire need of emphasis. The researcher believes that this KAP survey and the project as a whole will assist in developing a better understanding of the problem at hand, add to the knowledge base and research evidence and most importantly assist in improving quality care in the pre-hospital setting. The questionnaire consists of 46 questions (demographically information, true/false, likert scales, MCQ, etc.) and we are quite aware of the demand made on people to complete a survey of this nature. However, given the importance of the study for our profession and the quality of care we provide as well as the fact that it should take you no more than 20 minutes to complete, we sincerely hope that you will take the time to complete the survey. The outcomes of the research will be shared via Emergency Medical Services in the Western Cape.

Participation in this online survey is voluntary and there are no known or anticipated risks. This study has received formal ethical clearance from the Human Research Ethics Committee of the University of Cape Town. You may decline to participate or exit the survey at any time. Participants can withdraw until they submit the survey, at which point it will be anonymized. Should you wish to ask any further questions or require clarity on any aspect of the survey before making the decision whether to participate or not, please send an e-mail to andritl@gmail.com.

Sincerely

Andrit Lourens

1. Electronic Consent: Clicking on the **"agree"** button below indicates that

- You have read the above information
- You voluntarily agree to participate

If you do not wish to participate in the research study, please decline participation by clicking on the **"disagree"** button.

- ○ Agree
- ○ Disagree

Section 1: Demographic Information

The section on demographic information requires no personal identification information. Kindly complete.

2. Indicate your gender:

- ○ Male
- ○ Female

3. Indicate your age in years (numbers only) ___________________________

4. Indicate your highest clinical qualification related to the profession of Emergency Medical Care (EMC):

- ○ Basic Ambulance Assistant (BAA)
- ○ Ambulance Emergency Assistant (ANA)
- ○ Emergency Care Assistant (ECA)
- ○ Emergency Care Technician (ECT)
- ○ Critical Care Assistant (CCA) Paramedic
- ○ National Diploma in Emergency Medical Care (EMC)
- ○ B Tech in Emergency Medical Care (EMC) or Bachelor of Emergency Medical Care (BEMC)

5. Indicate the region in which you are currently practising:

- ○ Cape Town Metropolitan
- ○ Cape Winelands District
- ○ Central Karoo District
- ○ Eden District
- ○ Overberg District
- ○ West Coast District

6. Total years of experience in Emergency Medical Care or Emergency Medical Services (EMS) (years working in the pre-hospital setting, any qualification) _______________________

7. Current employment within Emergency Medical Services (EMS), indicate the most appropriate option for main function/responsibility:

- O Operational Emergency Care Provider
- O Supervisor / Manager
- O Education (Further Education and Training)
- O Education (Higher Education)
- O Rescue
- O Continuous Quality Improvement / Patient Safety
- O Communications
- O Student (Emergency Medical Care)
- O NOT currently working within EMS (please specify) __________________

8. Indicate sector of current employment:

- O Public/Government
- O Sector Private Sector
- O Other (please specify) __________________

9. Did you receive continuous medical education (CME) on the topic, acute pain assessment and/or management in the last two (2) years?

- O Yes
- O No

Section 2: True/False

Evaluate the 18 statements below and select TRUE, FALSE or DON'T KNOW.

10. Vital signs are always reliable (good) indicators of the intensity or severity of a patient's pain.

- O True
- O False
- O Don't know

11. Due to an underdeveloped nervous system, children younger than 2 years, have decreased sensitivity to pain and limited memory of painful experiences.

- O True
- O False
- O Don't know

12. Adult and paediatric patients who can be distracted from their pain are usually not experiencing severe pain.

- ○ True
- ○ False
- ○ Don't know

13. Children younger than 11 years cannot reliably report pain, therefore, clinicians should rely solely on the parent's assessment of the child's pain intensity.

- ○ True
- ○ False
- ○ Don't know

14. Giving patient's sterile water by injection (placebo) is a useful test to determine if their pain is real.

- ○ True
- ○ False
- ○ Don't know

15. If the source of a patient's pain is unknown, opioids/narcotics (like morphine) should not be used during the pain evaluation period, as this could mask the ability to correctly diagnose the cause of pain.

- ○ True
- ○ False
- ○ Don't know

16. Pain can be defined as "An unpleasant sensory and emotional experience associated with actual or potential tissue damage or described in terms of such damage."

- ○ True
- ○ False
- ○ Don't know

17. Similar or comparable stimuli, in different people, will produce the same intensity or severity of pain.

- ○ True
- ○ False
- ○ Don't know

18. Young infants, less than 6 months of age, cannot tolerate opioids/narcotics (like morphine) for pain relief.

- O True
- O False
- O Don't know

19. Non-pharmacological methods, such as splinting, are effective methods to assist pain relief.

- O True
- O False
- O Don't know

20. In the event that a patient's pain is not managed, their overall clinical condition may deteriorate (progressively worsen).

- O True
- O False
- O Don't know

21. Entonox® (Nitrous Oxide) is a potent analgesic with a very rapid onset of action and is quickly eliminated from the body.

- O True
- O False
- O Don't know

22. Unconscious patients do not experience pain.

- O Yes
- O No
- O Don't know

23. The experience and expression of pain are influenced by a patient's culture and/or spiritual beliefs.

- O Yes
- O No
- O Don't know

24. If you do not consider the condition to be painful the patient should not receive analgesia (pain relief).

- O True
- O False
- O Don't know

25. Patient behaviour is a more reliable (good) indicator of pain than a patient's self-report.

- True
- False
- Don't know

26. In the pre-hospital environment, patients should not receive analgesia for chronic medical conditions.

- True
- False
- Don't know

27. Self-reports of pain according to the numeric rating scale (pain assessment tool) are the quickest way to assess pain.

- True
- False
- Don't know

Section 3: Likert-scale

Please review the following 8 statements and select based on your opinion whether you agree, neither agree or disagree or disagree with the statements on the provided scale.

28. It is better to be stoic (endure pain or hardship without showing their feelings or complaining) about pain than totally open about it.

- Agree
- Neither agree nor disagree
- Disagree

29. Using a pain assessment tool is a necessary instrument in pain assessment and pain management decision making.

- Agree
- Neither agree nor disagree
- Disagree

30. I believe that my prior experience dealing with patients in pain allows me to score patients' pain more accurately than the patient themselves.

- Agree
- Neither agree nor disagree
- Disagree

31. The main reason for administering analgesia (pain relief) is to enable the patient to get to the ambulance.

- O Agree
- O Neither agree nor disagree
- O Disagree

32. Parents or guardians of children should not be present during * painful procedures.

- O Agree
- O Neither agree nor disagree
- O Disagree

33. Patients should not be included in the pain management decision-making process.

- O Agree
- O Neither agree nor disagree
- O Disagree

34. Expectations of my peers or the company/EMS service I work for, strongly influence my pain management practice.

- O Agree
- O Neither agree nor disagree
- O Disagree

35. The current HPCSA protocols provide sufficient and appropriate options for pain management in the pre-hospital setting in South Africa.

- O Agree
- O Neither agree nor disagree
- O Disagree

Section 4: Multiple Choice Questions

Review the 5 multiple choice questions below and select the most appropriate answer to the questions.

36. The correct wording when using the Numeric Rating Scale is:

- O Can you give your pain a score between 0 and 10 with zero (0) being no pain and 10 the worst imaginable pain?
- O Can you give your pain a score between 0 and 10 with 10 being no pain and zero (0) the worst imaginable pain?
- O Can you give your pain a score between 1 and 10 with zero (0) being no pain and 10 the worst imaginable pain?

- ○ Can you give your pain a score between 1 and 10 with 10 being no pain and zero (0) the worst imaginable pain?

37. The most accurate judge of the intensity of the patient's pain is:

- ○ The treating doctor
- ○ The emergency care provider
- ○ The patient
- ○ The patient's spouse, parent or family

38. Pain is believed to play a major part in the activation of the 'stress' response to injury, leading to all of the below, EXCEPT:

- ○ Increase in sympathetic nervous system activity
- ○ Impair immune function
- ○ Decreased coagulability
- ○ Catabolic hormone release

39. Effective management of acute pain is a fundamental component of:

- ○ Clinical documentation
- ○ Continuous care
- ○ Quality patient care
- ○ Emergency care

40. With regards to pain, all of the following descriptors are applicable EXCEPT:

- ○ Always subjective
- ○ Often undertreated
- ○ Always associated with actual tissue damage
- ○ A primary reason patient seeks medical advice

Section 5: Barriers and Enablers

41. Barriers to Pain Assessment and Management: In your opinion which of the below are possible barriers to (obstacle to or preventing) effective pain assessment and management in your previous and/or current work environments (select all that apply and please add any additional not listed in the box below).

- ○ Workload and lack of time
- ○ Service-related standard operating procedures or policy
- ○ Culture in the emergency service or work environment
- ○ Lack of available clinical practice guidelines to guide decision making

- ○ Unfamiliarity with protocols, medications or indications for pain management
- ○ Lack of resources (medications, disposables, nasal atomizers, etc.) to manage pain
- ○ Insufficient availability of clinical education
- ○ Practitioners reluctance to administer medication to manage pain
- ○ Concerns about causing more pain
- ○ Concerns about adverse effects secondary to analgesic agents
- ○ Difficulty to calculate medication dosages
- ○ Difficulty to assess pain
- ○ Uncooperative patient
- ○ Inability to determine adequate history/allergies
- ○ Patient spiritual, cultural or religious believes
- ○ Paediatric patients
- ○ Parental influence or involvement
- ○ Patient reluctance to report pain
- ○ Patient alcohol or drug use
- ○ Patient reluctance to receive analgesic agents
- ○ Language
- ○ Other (add any other barriers) ____

42. Enablers to Pain Assessment and Management: Enablers of Pain Assessment and Management: In your opinion, which of the below are possible enablers (allows achievement) of effective pain assessment and management in your work current and/or previous environment (select all that apply and please add any additional not listed in the box below).

- ○ Availability of higher qualified emergency care providers
- ○ Available clinical practice guidelines which guides decision-making
- ○ Service or company prioritize pain management
- ○ Service-related standard operating procedures or policy
- ○ Regular pain assessment facilitates good pain management
- ○ Supportive management and leadership structure with work environment or emergency service
- ○ Resources (medications, disposables, monitoring equipment) always available
- ○ Regular clinical education
- ○ Cooperative patients
- ○ Regular clinical audits
- ○ Pain management is important
- ○ Other (add any other enablers) ____

Section 6: Case Studies

Review the two case studies below. For each patient indicate your decision about the patient's pain scale and the management you would provide.

Andrew is 25 years old and this is his first day following abdominal surgery. As you enter his room, he smiles and continues talking and joking with his visitor. You are required to transport him to a hospital closer to home. Your assessment reveals the following information: BP = 120/80 mmHg; Heart Rate = 80 bpm; Respiratory Rate = 18 bpm. When questioned about his pain, on a scale of 0 to 10 (0 = no pain/discomfort, 10 = worst pain/discomfort) he rates his pain as 8.

43. On the patient record care report form, you are required to indicate his pain score. Select the number on the below scale that represents your assessment of Andrew's pain.
44. Indicate how you will manage Andrew's pain.

Robert is 25 years old and this is his first day following abdominal surgery. As you enter his room, he is lying quietly in bed and grimaces as he turns in bed. You are required to transport him to a hospital closer to home. Your assessment reveals the following information: BP = 120/80mmHg; Heart Rate = 80 bpm; Respiratory rate = 18 bpm. When questioned about his pain, on a scale of 0 to 10 (0 = no pain/discomfort, 10 = worst pain/discomfort) he rates his pain as 8.

45. On the patient record care report form, you are required to indicate his pain score. Select the number on the below scale that represents your assessment of Robert's pain.

46. Indicate how you will manage Robert's pain.
