# Supplementary material for: Acute pain assessment and management in the prehospital setting, in the Western Cape, South Africa: a knowledge, attitudes and practices survey
Source: BMC Emerg Med. 2020 Apr 28;20:31. doi: 10.1186/s12873-020-00315-0 (PMC7187518; doi:10.1186/s12873-020-00315-0)
Supplement: Supplementary file 2 — Additional file 2: Table S1: Comparing overall score between demographic groups, Table S2: Barriers to and enablers of pain assessment and management, Fig. S1 and S2: Pain scores for scenario - patient 1 (Andrew) and 2 (Robert), Table S3: Pain management for case scenarios. [file 12873_2020_315_MOESM2_ESM.pdf]

Additional File 2:

Table S1: Comparing overall score between demographic groups (n=87)

	Test Score	Mean Rank	p-value
**Gender***			
Male (n=59)	718.5	45.82	0.327
Female (n=28)		40.16	
**Highest Qualification****			
BLS (n=19)	30.79	20.79	<0.001
ILS (n=44)		43.31	
ALS (n=24)		63.65	
**Years' Experience****			
0-10 Years (n=50)	9.051	37.34	0.011
11-20 Years (n=30)		51.18	
21-30 Years (n=7)		60.79	
**Continuous Medical Education on acute pain assessment and management received in the last 2 years***			
Yes (n=46)	664.0	37.93	0.017
No (n=41)		50.80	
**Age Groups****			
20-30 Years (n=30)	2.800	38.00	0.424
31-40 Years (n=37)		46.07	
41-50 Years (n=18)		49.25	
51-60 Years (n=2)		48.50	
*Mann-Whitney U test (p value < 0.05, two-tailed), **Kruskal-Wallis H test (p value < 0.05, two-tailed)			

Table S2: Barriers to and enablers of pain assessment and management (n=73)

Barriers to pain assessment and management	n (%)
Patient alcohol or drug use	49 (67.1%)
Language	45 (61.6%)
Workload and lack of time	44 (58.9%)
Uncooperative patient	42 (57.5%)
Lack of resources (medications, disposables, nasal atomizers, etc.) to manage pain	37 (50.7%)
Patient spiritual, cultural or religious believes	34 (46.6%)
Inability to determine adequate history/allergies	33 (45.2%)
Paediatric patients	32 (43.8%)
Culture in the emergency service or work environment	25 (34.2%)
Practitioners reluctance to administer medication to manage pain	25 (34.2%)
Parental influence or involvement	24 (32.9%)
Lack of available clinical practice guidelines to guide decision making	22 (30.1%)
Difficulty to assess pain	22 (30.1%)
Patient reluctance to report pain	22 (30.1%)
Service-related standard operating procedures or policy	21 (28.8%)
Patient reluctance to receive analgesic agents	20 (27.4%)
Concerns about adverse effects secondary to analgesic agents	20 (27.4%)
Insufficient availability of clinical education	19 (26.0%)
Unfamiliarity with protocols, medications or indications for pain management	18 (24.7%)
Concerns about causing more pain	15 (20.5%)
Difficulty to calculate medication dosages	12 (16.4%)
Other: see additional items added below	4 (5.5%)
Insufficient number of Advanced Life Support practitioners,	
Protocol deficiency,	
Availability of ketamine (better analgesic medication for prehospital use than morphine),	
Lack of expertise,	
Entonox® not available on all ambulances	
**Enablers of pain assessment and management**	**n (%)**
Availability of higher qualified emergency care providers	54 (74.0%)
Pain management is important	43 (58.9%)
Resources (medications, disposables, monitoring equipment) always available	38 (52.1%)
Cooperative patients	38 (52.1%)
Regular clinical education	36 (49.3%)
Available clinical practice guidelines which guide decision making	32 (43.8%)
Regular pain assessment facilitates good pain management	28 (38.4%)
Service or company prioritise pain management	27 (37.0%)
Supportive management and leadership structure with work environment or emergency service	26 (35.6%)
Service-related standard operating procedures or policy	22 (30.1%)
Regular clinical audits	21 (28.8%)
Other: see additional items added below	3 (4.1%)
Regular updates,	
Providing all qualifications in emergency care with more efficient analgesic medications,	
Knowing the quality of pain ("stabbing versus burning pain")	

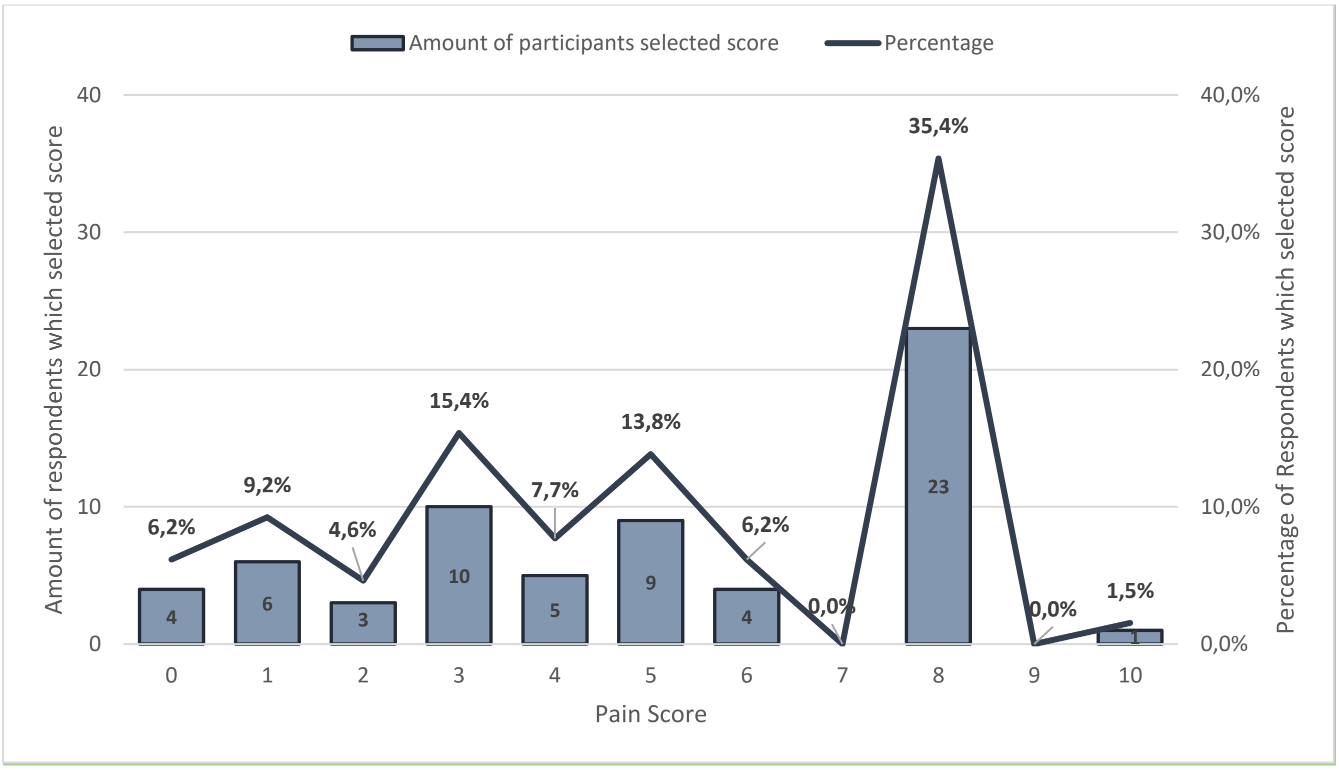

Figure S1: Pain score scenario – patient 1 (Andrew) (n=65)

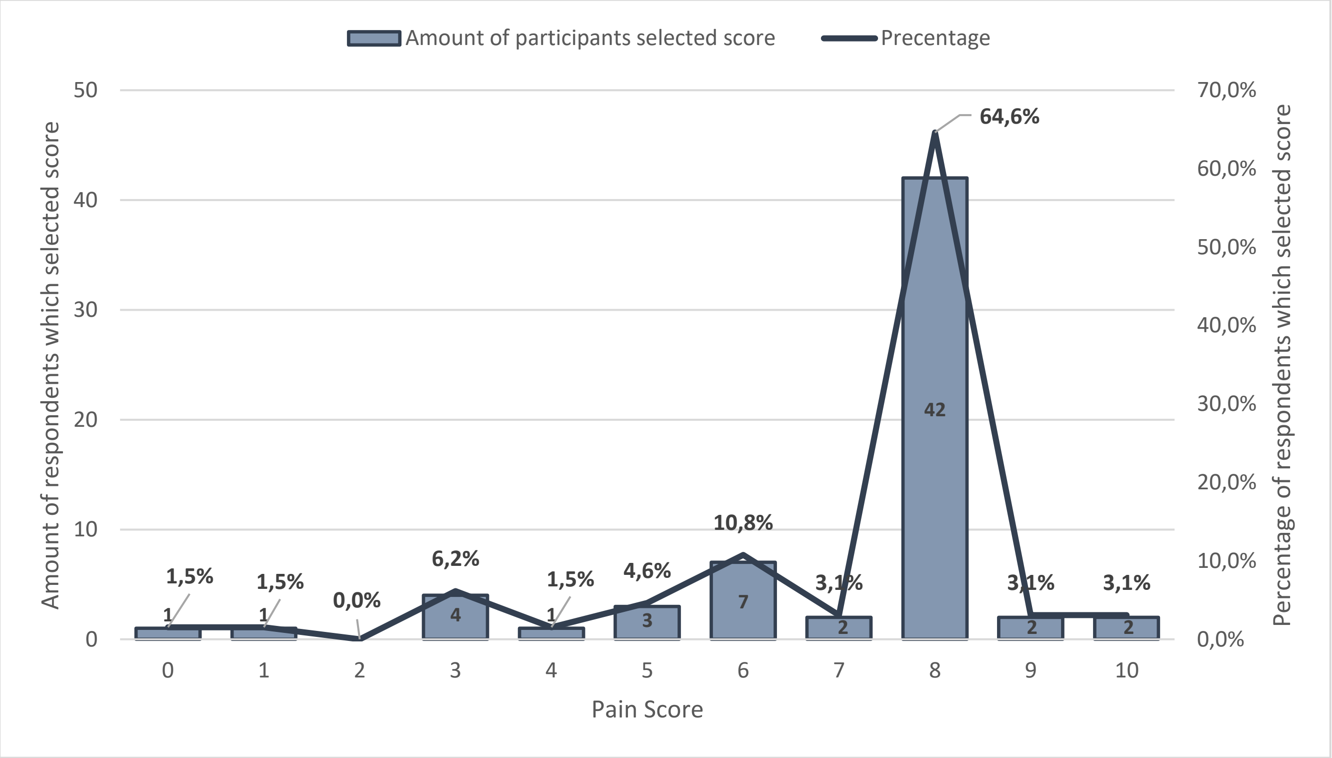

Figure S2: Pain score scenario – patient 2 (Robert) (n=65)

Table S3: Pain management for case scenarios (n=65)

Qualification	Patient 1 (Andrew's)	Patient 2 (Robert's)
**BLS (n=12)**	- Re-assess patient & conduct ongoing assessment<br>- Observe facial expressions<br>- Position & make patient comfortable, ask nurse to administer pain medication before transportation<br>- Administer 40% oxygen<br>- Administer Entonox® according to protocol	- Re-assess patient & conduct ongoing assessment<br>- "Calm patient"<br>- Position & make patient comfortable, ask nurse to administer pain medication before transportation<br>- Administer 40% oxygen<br>- Administer Entonox® according to protocol
**ILS (n=32)**	- Calm & re-assess the patient<br>- Position & make comfortable, monitor vital signs & transport<br>- Consider the administration of oxygen 40%<br>- Administer Entonox® according to protocol<br>- No pain medication on scope of practice, therefore, will start oxygen & IV therapy in case advanced life support (ALS) backup is needed to administer pain medication, make patient comfortable<br>- Request for the administration of pain medication from nursing staff<br>- Consult ALS practitioner	- Calm & re-assess the patient<br>- Position & make comfortable, monitor vital signs & transport<br>- Consider the administration of oxygen 40%<br>- Administer Entonox® according to protocol<br>- No pain medication on scope of practice, therefore, will start oxygen & IV therapy in case ALS backup is needed to administer pain medication, make patient comfortable<br>- Consult ALS practitioner or request backup for pain medication<br>- Request for the administration of pain medication from nursing staff
**ALS (n=21)**	**Emergency Care Technician (n=4)**	
	- Provide "Tender Loving Care (TLC)"<br>- Make patient comfortable & titrate pain medication<br>- Assess vital signs, obtain history (allergies) & administer pain medication if needed<br>- Conduct visual assessment (overall behaviour & facial expressions) as patient may by enduring pain or exaggerating pain. Administer placebo & reassess pain score, if the same transport.	- Provide "TLC" & pain medication<br>- Since patient in the hospital, consultant doctor regarding analgesia before departure & during transfer<br>- Assess vital signs, obtain history (allergies) establish intravenous (IV) therapy, administer pain medication & monitor<br>- Administer placebo & re-assess pain, in the event the pain score remains the same, consult with the medical officer
	**Paramedic (including Critical care Assistant and National Diploma in Emergency Medical Care paramedic) (n=9)**	
	- "Calm & reassure"<br>- "No intervention"<br>- Manage conservatively, position, if ineffective administer pain medication<br>- If the patient request, administer analgesia prior to loading the patient for transportation<br>- Position & make comfortable, monitor to see whether pain medication needed<br>- Administer morphine (3 mg) IV & monitor	- "TLC"<br>- "No intervention"<br>- Position, if pain did not take any pain medication, administer analgesia<br>- Administer analgesia prior to loading the patient for transportation<br>- Administer morphine (titrate 1 mg to the desired effect, 5mg) IV & monitor
	**Emergency Care Practitioner (n=8)**	
	- Comfort patient<br>- Reposition, if no pain relief administer analgesia<br>- Inquire whether the patient requires pain medication, administer 1g of paracetamol before considering opioids, would consider adverse effects of opioids in this patient before administration<br>- Ask the hospital for oral analgesia<br>- Administer penthrox (penthroxyflurane)<br>- Administer morphine<br>- Consider patient factors, moving patient will increase pain, inquire about whether pain medication was administered & when, inquire whether patient what pain medication & document replay, administer IV morphine but IV paracetamol would be preferred.	- Comfort patient<br>- Inquire whether something reliefs the pain, if not administer analgesia<br>- Inquire whether the patient requires pain medication, administer 1g of paracetamol IV before considering opioids, would consider adverse effects of opioids in this patient before administration<br>- Administer morphine IV or ketamine IV<br>- Consider patient factors & determine whether the patient is overstating pain since pain management is a vital part of patient care administer morphine & transport patient in a position of comfort.
